# Precise Construction of an Antimicrobial Peptide Targeting Bacterial Cell Membranes Derived From Natural Peptides

**DOI:** 10.1002/advs.202517068

**Published:** 2026-01-12

**Authors:** Jiaqi Huang, Bohao Liu, Xingzhuo Zhu, Deqian Qiao, Sizhe Chen, Xiaoyan Zeng, Qingqing Yang, Zihuan Wei, Yinjuan Huang, Jizhao Wang, Guangjian Zhang, Qiuyu Gong

**Affiliations:** ^1^ Department of Thoracic Surgery The First Affiliated Hospital of Xi'an Jiaotong University Xi'an P. R. China; ^2^ Key Laboratory of Enhanced Recovery After Surgery of Integrated Chinese and Western Medicine Administration of Traditional Chinese Medicine of Shaanxi Province The First Affiliated Hospital of Xi'an Jiaotong University Xi'an P. R. China; ^3^ Microbiota I‐Center Chinese University of Hong Kong Hong Kong P. R. China; ^4^ Department of Laboratory Medicine The First Affiliated Hospital of Xi'an Jiaotong University Xi'an P. R. China; ^5^ State Key Laboratory of Porous Metal Materials Shaanxi International Research Center for Soft Matter School of Materials Science and Engineering Xi'an Jiaotong University Xi'an P. R. China; ^6^ Key Laboratory of Optic‐Electric Sensing and Analytical Chemistry For Life Science Ministry of Education Qingdao University of Science and Technology Qingdao P. R. China

**Keywords:** amino acid mutation, antimicrobial peptide, insect cuticle

## Abstract

Antimicrobial peptides (AMPs) are promising alternatives to overcome antimicrobial resistance (AMR). However, precise construction of an AMP targeting bacterial cell membranes derived from natural peptides remains a great challenges. Although the artificial intelligence (AI) algorithm‐assisted screening method has achieved unprecedented successes, it's difficult to predict the targets of AMPs obtained from this method. To address this, an AMP (**P 3‐3R‐8I**) based on several natural peptides derived from insect cuticle was constructed precisely via amino acid mutation. The mutated amino acids Arginine (R) and Isoleucine (I) are expected to target the bacterial cell membranes. Surprisingly, **P 3‐3R‐8I** exhibits super antibacterial capability against two representative bacteria: methicillin‐resistant *Staphylococcus aureus* (MRSA) and *Escherichia coli* (*E. coli*), which could be attributed to the ability to quickly penetrate bacterial cell membranes and then to bind to bacterial DNA of **P 3‐3R‐8I**, resulting in the suppression of DNA replication. In rats’ model, the MRSA‐infected wound could be alleviated by **P 3‐3R‐8I** obviously, as well as lung and spleen infections in MRSA‐induced systemic sepsis. Our findings provide a prospect for the precise construction of AMPs targeting bacterial cell membranes as well as a means of overcoming AMR, offering a strategy for drug‐resistant bacteria‐induced tissue repair.

## Graphical abstract

An AMP (**P 3‐3R‐8I**) based on natural peptides, which can target bacterial cell membranes, was precisely constructed via amino acid mutation. **P 3‐3R‐8I** exhibits antibacterial capability which could be attributed to the ability of **P 3‐3R‐8I** to quickly penetrate bacterial cell membranes and then to bind to bacterial DNA. In vivo, the MRSA‐infected wound and systemic sepsis in rats could be repaired by **P 3‐3R‐8I**.

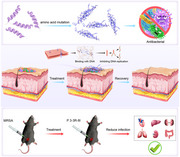

## 1 Introduction

Antimicrobial resistance (AMR) has become a serious global public health issue due to the misuse of antibiotic [1, 2, 3, 4]. Alternatively, nano‐materials offer opportunities to overcome AMR with effective interference with the bacterial systems and augmentation of biofilm penetration [5]. Unfortunately, the clinical applications or translations of nano‐materials for AMR are limited by their unclear biosafeties, inhomogeneities, and cost‐effectiveness [6, 7]. Unlike nano‐materials, antimicrobial peptides (AMPs), evolutionarily ancient and multifunctional macromolecules, are promising candidates to address AMR [8, 9, 10, 11]. Although the screening and identification of an AMP remains great challenges, artificial intelligence (AI) algorithm‐assisted screening method has achieved unprecedented successes. [12, 13, 14, 15, 16, 17, 18] However, it is very difficult to precisely predict the targets of AMPs obtained from AI algorithm‐assisted screening method. Hence, to address this, amino acid mutation method arises our interest.

Amino acid mutations are usually employed to investigate the functions of peptides. [19] For AMPs, the antibacterial properties could be regulated via the mutations of some key amino acid residues. Liu et al. screened an AMP (AMP 1) based on AI method, and found that substitution of Tryptophan (W) with Leucine (L) in AMP 1 leads to the weakening of the bacterial cell membrane penetration ability of AMP 1, resulting the decline of its antibacterial performance. [8] Kim et al. suggested that the membrane penetration abilities of AMPs could be enhanced via reasonable mutations or substitutions of cationic amino acid residues, where the activities of AMPs were improved. [20] Held et al. developed a deep mutational scanning platform for the adjustment of amino acid residues to control the antibacterial capacity of AMPs. 21 Despite this, the precise construction of an AMP targeting bacterial cell membranes derived from natural peptides and its applications in drug resistant bacteria‐induced tissue repair are still less reported.

In this study, we aim to obtain an AMP targeting bacterial cell membranes. With the amino acid mutation method, an AMP (named **P 3‐3R‐8I**) based on several natural peptides derived from insect cuticle was constructed precisely. Based on the W contents, percentages of hydrophobic and positive charged amino acid residues, five natural peptides (**P 1**‐**P 5**) from an *Ostrinia furnacalis* cuticular protein were classified. Among them, several uncharged or negative charged as well as weak hydrophobic residues in **P 3** and **P 4** were replaced by positively charged (mainly Arginine‐R) or strong hydrophobic residues (mainly Isoleucine‐I). R and I are expected to enhance the targeting capacity as well as the penetration performances of **P 3‐3R‐8I** for bacterial cell membranes. Interestingly, **P 3‐3R‐8I** exhibits super antibacterial capability against two representative bacteria: methicillin‐resistant *Staphylococcus aureus* (MRSA) and *Escherichia coli* (*E. coli*, ATCC 25922), which could be attributed to the ability of **P 3‐3R‐8I** to quickly penetrate bacterial cell membranes and then to bind to bacterial DNA, resulting in the suppression of DNA replication. In vivo, the MRSA‐infected wound could be alleviated by **P 3‐3R‐8I** obviously, as well as lung and spleen infections in MRSA‐induced systemic sepsis. Our findings highlight a prospect for precise construction of AMPs targeting bacterial cell membranes based on natural peptides as well as a means of overcoming AMR, offering a strategy for drug‐resistant bacteria‐induced tissue repair.

## 2 Results and Discussion

### 2.1 Construction of an AMP: P 3‐3R‐8I

**FIGURE 1:**
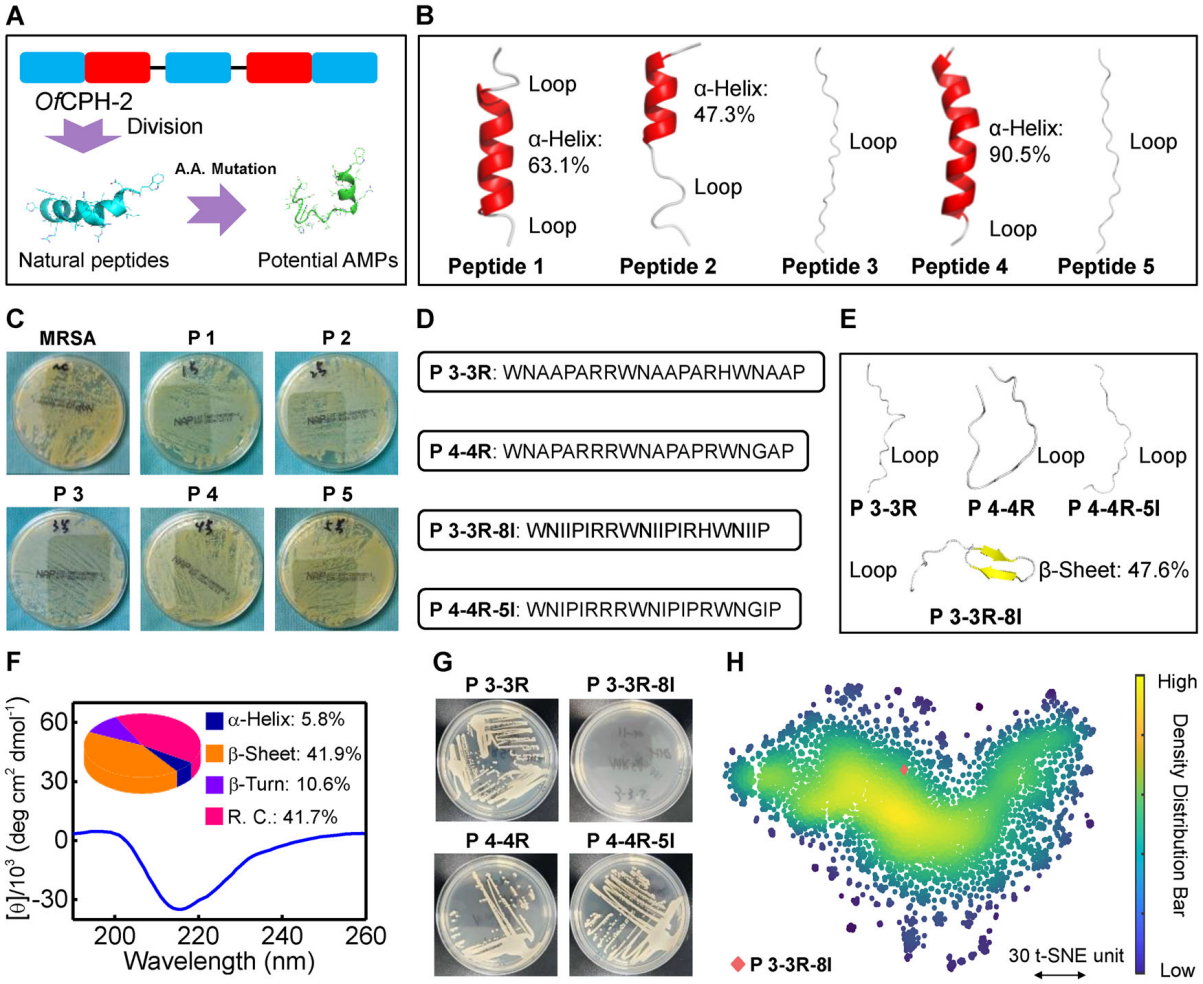
Construction of an AMP via amino acid mutation. (A) The schematic diagram of the construction process of potentail AMPs. (B) The structures of peptide 1 (**P 1**) to **P 5** predicted by AlphaFold3. (C) The representative photos of MRSA colonies treated with **P 1** to **P 5** (250 µM) for 24 h or without peptides. n = 3. (D) The amino acied squences of mutated peptides: **P 3‐3R**, **P 4‐4R**, **P 3‐3R‐8I,** and **P 4‐4R‐5I**. (E) The structures of **P 3‐3R**, **P 4‐4R**, **P 3‐3R‐8I,** and **P 4‐4R‐5I** predicted by AlphaFold3. (F) The CD spectrum of **P 3‐3R‐8I** (250 µM, pH 7.4) and the contents of secondary structures calculated using BESTSEL. (G) The representative photos of MRSA colonies treated with **P 3‐3R**, **P 4‐4R**, **P 3‐3R‐8I,** and **P 4‐4R‐5I** (peptide concentrations: 250 µM) for 24 h. n = 3. (H) The t‐SNE plot of **P 3‐3R‐8I** and other known AMPs.

Our group has found that *Of*CPH‐2 is an excellent antibacterial protein for wound management [22]. However, the targeting performance of *Of*CPH‐2 is unclear, and the bioavailability of *Of*CPH‐2 may be hindered by its large molecular weight (∼26.2 kDa), hence, construction of AMPs targeting bacterial cell membranes with a lower molecular weight is necessary. By analyzing the sequence of one previous antibacterial protein (*Of*CPH‐2) (22), 18aa (commonly existed in some insects [23], red marked in Figure S1) and five natural peptides were divided (blue marked in Figure S1), which was named as peptide 1 to 5 (abbreviated as **P 1** to **P 5**, some basic information were shown in Figures S2–S6 and Table S1). And the construction process of potential AMPs was shown in Figure 1A. **P2**, **P3**, **P4,** and **P5** have similar sequence distributions. And it can be seen that the main structures of **P 1**, **P 2,** and **P 4** were predicted as α‐helix (Figure 1B), while **P 3** and **P 5** exhibited loop‐like ones (Figure 1B). In addition, it is reported that W plays critical role in antimicrobial activity due to that the bacteria cell membrane penetration abilities of AMPs could be enhanced by W, [24] and the W contents (the ratio of the number of W to the total number of amino acids) are kept high (>5%) in both five peptides (Figure S7), which is beneficial for antibacterial properties. Among them, the W contents of **P 3** (14.28%) and **P 4** (15.00%) were higher than those of other amino acids (Figure S7), indicating the existence of possible antimicrobial activities. Meanwhile, the high ratio of hydrophobic amino acid in **P 3** (38.09%, Figure S7) suggested that there exists strong interactions between peptide and lipids in bacteria cell membrane. [25 Lastly, positively charged residues seems to enhance the lipopolysaccharide or lipoteichoic acid‐AMPs/phosphatidylglycerol or phosphatidylserine‐AMPs interactions, resulting in bacteria death. [26, 27] The low contents of positively charged residues in **P 1**, **P 3** and **P 4** endowed the transformation capacity of them to AMPs. Howerer, **P 1** to **P 5** exhibited no inhibition effets on MRSA or *E. coli* (ATCC 25922) (Figure 1C; Figure S8), and most AMPs are amphiphilic molecules, [28 hence, the lack of antimicrobial activities of **P 1** to **P 5** may be attributed to the fact that none of them were obviously amphiphilic molecules (Figure S9).

Furthermore, we chose **P 3** and **P 4** for amino acid mutation to obtain potential AMPs. Proline (P) residue at position 7, Serine (S) residue at position 8 and Asparagine (N) residue at position 15 in **P 3** were mutated as R residue (**P 3‐3R**, Figure 1D; Figure S10 and Table S2), and Glutamine (Q) residue at positions 6–8 and 15 were mutated as R residues (**P 4‐4R**, Figure 1D; Figure S11 and Table S2) to heighten the contents of the positively charged residues (Figure S12). Besides, the Alanine (A) residues at position 3, 4, 6, 11, 12, 14, 19 and 20 in **P 3‐3R**, and residues at position 3, 5, 11, 13 and 19 in **P 4‐4R** were mutated as I residues (**P 3‐3R‐8I** and **P 4‐4R‐5I**, Figure 1D; Figures S13–S15 and Table S2 Supporting Information) to enhance the hydrophobicities of peptides and the bacterial cell membranes targeting performances. The main structures of **P 3‐3R**, **P 4‐4R,** and **P 4‐4R‐5I** were predicted as loop‐like ones (Figure 1E), while the **P 3‐3R‐8I** tend to be like β‐sheet stucture (Figure 1E, β‐sheet: 47.6%), which was further confirmed by circular dichroism (CD) spectral results (Figure 1F). Interestingly, it is generally believed that many AMPs predominantly adopts a β‐sheet structure, [29], and **P 3‐3R‐8I** showed a typical amphiphilic feature (Figure S16). These two properties made it as a potential AMP. As expected, **P 3‐3R‐8I** exhibited excellent antibacterial activity toward MRSA (model bacteria here, Figure 1G). To represent the intrinsic relationships between **P 3‐3R‐8I** within particular peptides, the statistical model of t‐distributed stochastic neighbor embedding (t‐SNE) was used. It is shown that **P 3‐3R‐8I** belonged to a type of AMPs (Figure 1H). All these results indicated that an AMP was successful constructed via amino acid mutation without complex arithmetic, a huge amount of training datasets, and long training time.

### 2.2 The Antibacterial Performances of P 3‐3R‐8I

**FIGURE 2:**
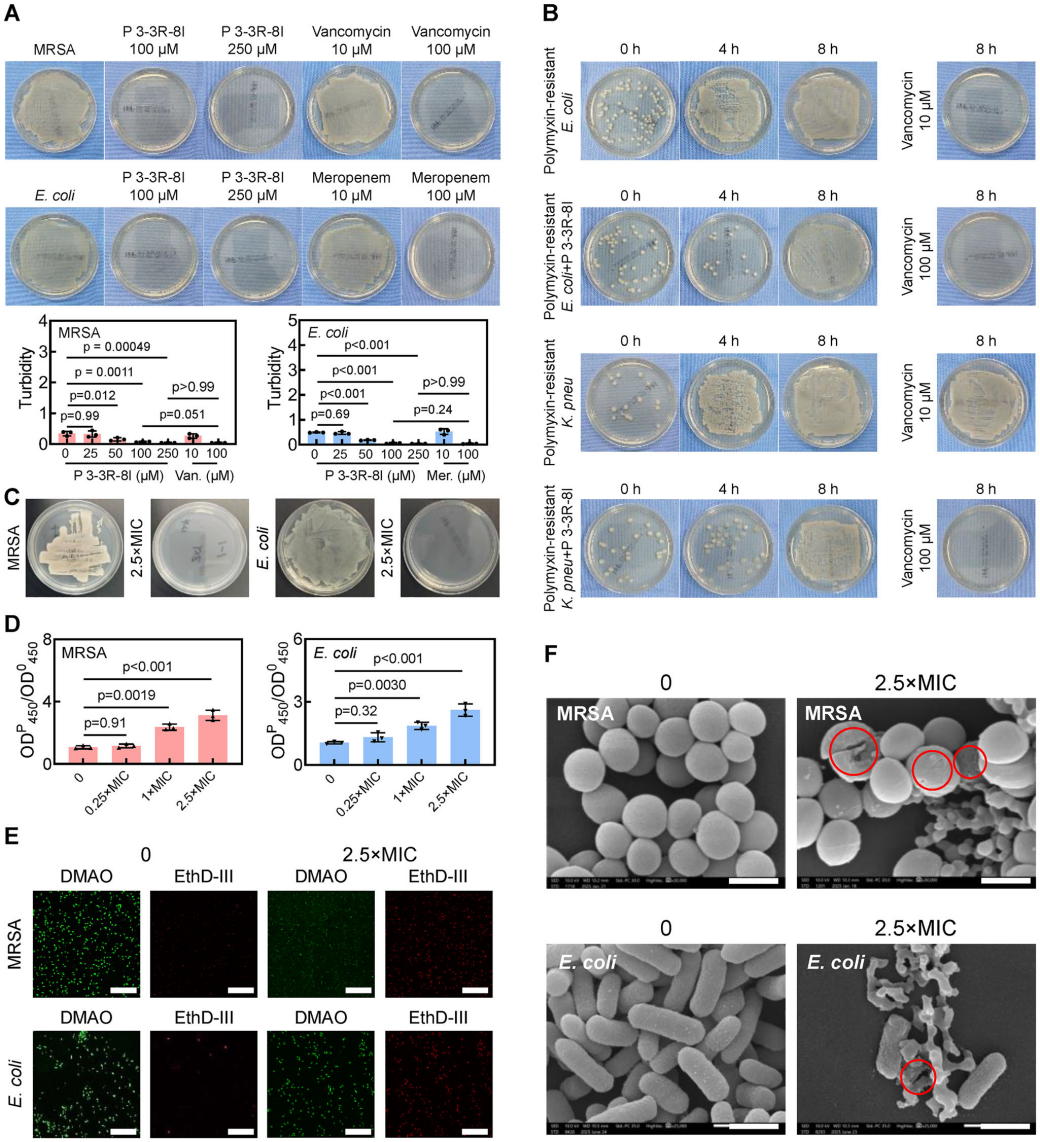
The antibacterial performances of **P 3‐3R‐8I**. (A) The representative photos of MRSA or *E. coli* (ATCC 25922) colonies treated with different concentrations of **P 3‐3R‐8I**, Vancomycin, and Meropenem. And the turbidities of bacterial liquids treated with different concentrations of **P 3‐3R‐8I**. n = 3. (B) The representative photos of polymyxin‐resistant *E. coli and* polymyxin‐resistant *K. pneu* colonies treated with **P 3‐3R‐8I** (250 µM) for different time or treated with different concentrations of Vancomycin for 8 h. n = 3. (C) The representative photos of MRSA or *E. coli* (ATCC 25922) colonies treated with 2.5×MIC of **P 3‐3R‐8I** for 24 h. n = 3. (D) The ratios of the aborbance at 450 nm of bacterial liquids treated with **P 3‐3R‐8I** to the corresponding aborbance at 450 nm of bacterial liquids. n = 3. (E) The representative fluorescent images of MRSA or *E. coli* (ATCC 25922) treated without or with **P 3‐3R‐8I** for 24 h stained with DMAO (green) and EthD‐III (red). Scale bar: 50 µm. n = 3. (F) The representative SEM images of MRSA or *E. coli* (ATCC 25922) treated without or with **P 3‐3R‐8I** for 24 h. n = 3. Scale bar: 500 nm.

Now that **P 3‐3R‐8I** has been identified as an AMP, its antibacterial performances will be studied systematically. As shown in Figure 2A, with an increase of **P 3‐3R‐8I** contentration, the MRSA colonies disappeared, which was observed in the group treated with Vancomycin (100 µM, positive drug). The same phenomenon was also observed in the example of *E. coli* (ATCC 25922) (Figure 2B, Meropenem as positive drug). Meanwhile, the turbidities of bacterial liquids were decreased (from ∼0.5 to 0.04) with the increase of **P 3‐3R‐8I** contentration (Figure 2A), and it can be concluded that the minimum inhibitory concentrations (MIC) of **P 3‐3R‐8I** toward MRSA and *E. coli* (ATCC 25922) were 100 µM. In addition, the effects of incubation time of **P 3‐3R‐8I** on the survivals of MRSA and *E. coli* were studied.). It can be seen that the MRSA colonies disappeared after the treatment with **P 3‐3R‐8I** for 8 h, and *E. coli* colonies disappeared after the treatment with **P 3‐3R‐8I** for 12 h (Figure S17). Moreover, the antibacterial abilities of AMPs against mobile colistin resistance‐positive (MCR+) strains is one of the important indicators to measure their performances, so the inhibition effects of **P 3‐3R‐8I** on two MCR+ strains: polymyxin‐resistant *E. coli and* polymyxin‐resistant Klebsiella pneumoniae (*K. pneu*) (Figure S18) were evaluated. As shown in Figure 2B, the MCR+ strains could be suppressed by **P 3‐3R‐8I** for 4 h, while this effect was weakened/disappeared with the incubation time, which may be attributed to that the target of **P 3‐3R‐8I** was not the drug‐resistance gene such as MCR‐1. The structures or sequences of **P 3‐3R‐8I** would be modified for mcr‐positive strains in our future work. Two MCR‐ strains (MRSA and *E. coli*, Figure S18) were used for the following studies (Figure 2C).

In order to determine the integrities of bacterial cell membranes, o‐nitrophenyl‐β‐d‐galactopyranoside (ONPG) assay was performed. [30] It can be seen that, the absorbance at 450 nm of solutions increased (from ∼1 to 3 or 4) with the **P 3‐3R‐8I** (Figure 2D), suggesting the presence of β‐galactosidase leaked from dead bacteria. The destorys of bacterial cell membranes were further confirmed by the live/dead staining results, in which enhanced red fluorescence indicates the death of bacteria (Figure 2E). Lastly, scanning electron microscope (SEM) results exhibited that some bacterial cell membranes were damaged (marked by red circle, Figure 2F) after treated with **P 3‐3R‐8I**, confirming the death of bacteria. These results show that **P 3‐3R‐8I** show excellent antibacterial performance. It shoud be noted that the interactions of between AMPs and bacterial cell membranes would lead to the release of intra‐bacterial substances, resulting in bacterial death. [31] However, the conclusion that the detriments of bacterial cell membranes were induced by **P 3‐3R‐8I** directly was not fully supported by the above results (Figure 2D‐F). Then, the main mechanism for bacterial death caused by **P 3‐3R‐8I** will be discussed afterward.

### 2.3 The Antibacterial Mechanism of P 3‐3R‐8I

**FIGURE 3:**
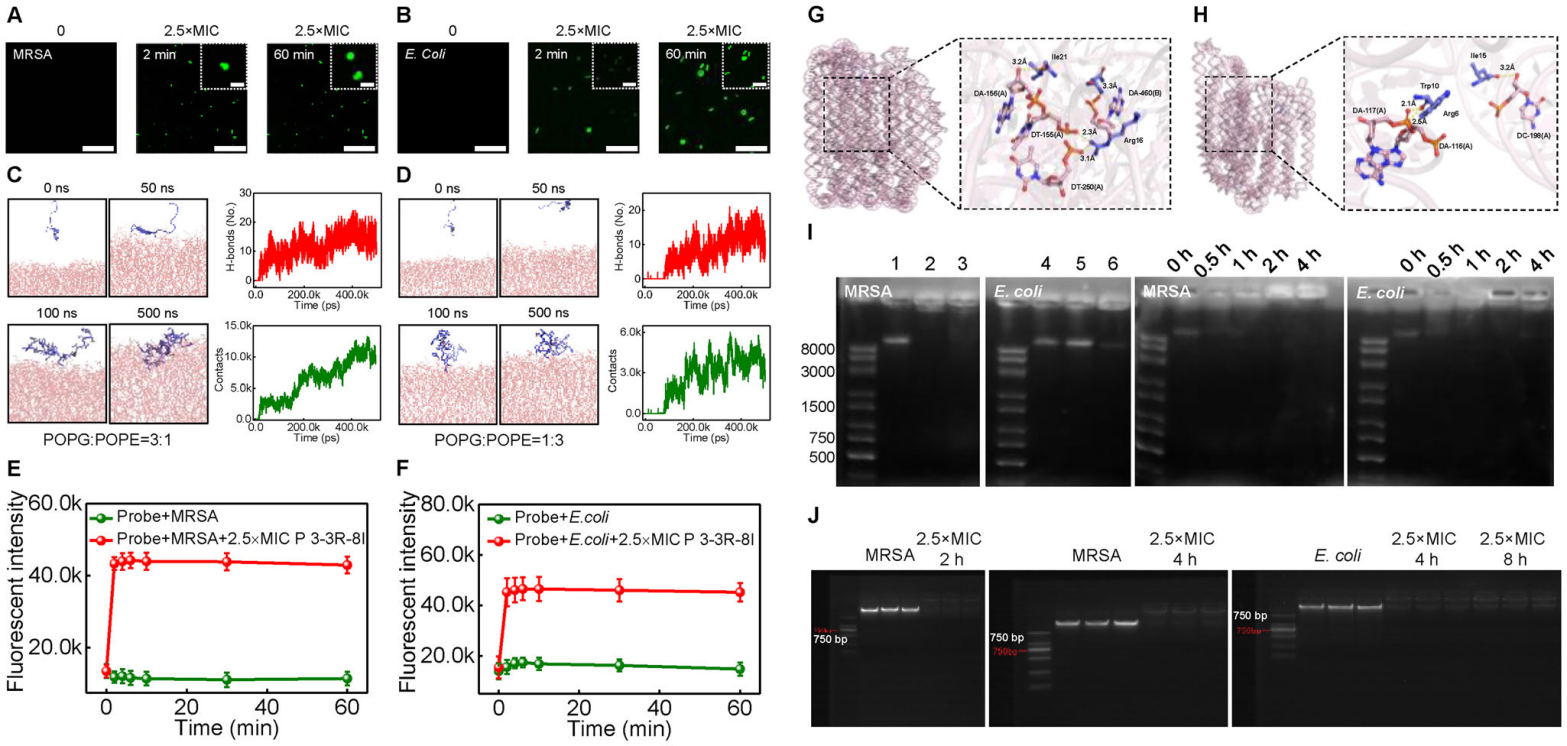
The antibacterial mechanism of **P 3‐3R‐8I**. (A,B) The representative confocal fluorescence images of MRSA or *E. coli* (ATCC 25922) treated with **P 3‐3R‐8I** for different time. n = 3. Scale bar: 50 µm for low multiple images. Scale bar: 10 µm for inset images. (C) The MD simulation processes of **P 3‐3R‐8I**‐gram‐positive bacterial‐mimicking membranes and the number of hydrogen bonds and contact atoms between them during MD simulation processes. (D) The MD simulation processes of **P 3‐3R‐8I**‐gram‐negative bacterial‐mimicking membranes and the number of hydrogen bonds and contact atoms between them during MD simulation processes. (E,F) The fluorescence of DiSC3(5) probe (5 µM) from different groups with time. n = 3. (G,H) The docking models between **P 3‐3R‐8I** and *Staphylococcus aureus*s’ DNA/*E. coli*s’ DNA. (I) The representative EMSA results of *Staphylococcus aureus*s’ DNA/E. colis’ DNA treated without or with **P 3‐3R‐8I**. 1: 0, 2: 0.25×MIC, 3: 2.5×MIC, 4: 0, 5: 0.25×MIC, 6: 2.5×MIC, time 1 h. For time dependent results, the concentration of **P 3‐3R‐8I** is 2.5×MIC. 37°C. n = 3. (J) The representative gel electrophoresis results of MRSA or *E. coli* (ATCC 25922) treated with **P 3‐3R‐8I** for different time. 37°C. n = 3.

The mutation principle that we mentioned is to precise construct the bacterial cell membranes targeting performacnes of AMPs, and interestingly, our above results indicated that the detriments of bacterial cell membranes were not caused by **P 3‐3R‐8I** directly. So the mechanism for bacterial death caused by **P 3‐3R‐8I** (labeled with fluorescein Isothiocyanate‐FITC, Figure S19) was studied. As shown in Figure 3A,B, **P 3‐3R‐8I** could target the bacterial cell membranes and penetrate them quickly (∼ 2 min), confirming the reliability of our mutation principle. To further understand the action modes of **P 3‐3R‐8I** with bacterial‐mimicking membrane (POPG:POPE = 3:1 represents gram‐positive bacterial membrane and POPG:POPE = 1:3 represents gram‐negative bacterial membrane), [32] the molecular dynamics (MD) simulations were performed. During 0–50 ns MD simulations, **P 3‐3R‐8I** contacts with gram‐positive bacterial‐mimicking membranes, and during 100–500 ns MD simulations, **P 3‐3R‐8I** are stably embedded in bacterial membranes (Figure 3C). More than 10 hydrogen bonds were formed between **P 3‐3R‐8I** and the membranes (Figure 3C), and van der Waals interactions have also been shown to exist (Figure S20A). Moreover, the number of contact atoms **P 3‐3R‐8I** and the gram‐positive bacterial‐mimicking membranes was more than 5000 (Figure 3C). At the same time, a similar phenomenon also existed in the interaction model between polypeptides and gram‐negative bacterial‐mimicking membranes (Figure 3D; Figure S20B). In addition, the root mean square deviations (RMSD) of **P 3‐3R‐8I** in gram‐positive bacterial‐mimicking membrane and gram‐negative bacterial‐mimicking membrane systems were about 0.5 nm (Figures S21 and S22). The root mean square fluctuation (RMSF) values of amino acid residues in both N‐ and C‐terminals were volatile (Figures S21 and S22), indicating the residues in both N‐ and C‐terminals were regulated to facilitate the interactions between **P 3‐3R‐8I** and membranes. The radius of gyration (Rg) were changed a littile after during MD simulation, implying the stability of **P 3‐3R‐8I** (Figures S21 and S22). Lastly, the contact surface areas (CSA) in both systems were above 10 nm^2^ (Figures S21 and S22). Importantly, in order to further confirm the interactions between **P 3‐3R‐8I** and bacterial cell membranes, the 3,3′‐dipropylthiacarbocyanine (DiSC3(5)) fluorescent membrane probe assay was performed. The DiSC3(5) probe would be accumulated in the lipid bilayer, resulting in the fluorescence quenching. If the membrane potential was influenced by AMPs, the probe would be released, and the fluorescent intensities were enhanced. [33] As shown in Figure 3E,F, the fluorescences of DiSC3(5) increased quickly upon treatment with **P 3‐3R‐8I** (∼2 min), indicating existence of the interactions between **P 3‐3R‐8I** and bacterial cell membranes. All these results show that the strong interactions between both gram‐positive and negative bacterial membranes and **P 3‐3R‐8I** can be formed quickly, and the **P 3‐3R‐8I** can be embedd into bacterial membranes stably.

As mentioned above, the interactions between **P 3‐3R‐8I** and bacterial cell membranes were not the main reason for antibacterial, hence, the effect of **P 3‐3R‐8I** on bacterial intracellular vitality was studied through a DNA‐binding assay. The binding model between **P 3‐3R‐8I** and bacterial DNA was first studied. As shown in Figures 3G the hydrophobic interactions existed between the amino acid residues of I6, W1, I12, I19, P21, I3, W17, I14, and P5 in **P 3‐3R‐8I** and *Staphylococcus aureus*s’ DNA. And the hydrogen bonds existed between the residues in **P 3‐3R‐8I** and *Staphylococcus aureus*s’ DNA (Table S3). Moreover, the hydrophobic interactions existed between the amino acid residues of I12, P13, W1, and W17 in **P 3‐3R‐8I** and *E. coli*s’ DNA (Figure 3H), and the hydrogen bonds also existed (Table S4). The docking score of **P 3‐3R‐8I** and *Staphylococcus aureus*s’ DNA/**P 3‐3R‐8I** and *E. coli*s’ DNA docking modes were ‐670.84 and ‐600.77, respectively, demonstrating the interactions between them were strong. Second, an electrophoretic mobility shift assay (EMSA) was conducted to further confirm the presence of binding between **P 3‐3R‐8I** and bacterial DNA. [34] As shown in Figure 3I, **P 3‐3R‐8I** exhibited high binding affinity to the genomic DNA of MRSA and *E. coli* (ATCC 25922), suggesting a possible bactericidal activity through interaction with bacterial DNA and interfering with the replication/transcription processes. Lastly, the gel electrophoresis assays were performed to study the effects of **P 3‐3R‐8I** on the replication performances of DNA. As shown in Figure 3J, the intensities of DNA bands were weakened to disappear upon the treatment of **P 3‐3R‐8I**, indicating DNA replications were suppressed by **P 3‐3R‐8I**. All these results exhibit that the antibacterial mechanism of **P 3‐3R‐8I** can be attributed to the ability to quickly penetrate bacterial cell membranes and then to bind to bacterial DNA of **P 3‐3R‐8I**, resulting in the suppression of DNA replication.

### 2.4 The Application of P 3‐3R‐8I in MRSA‐Infected Wound Healing Management

**FIGURE 4:**
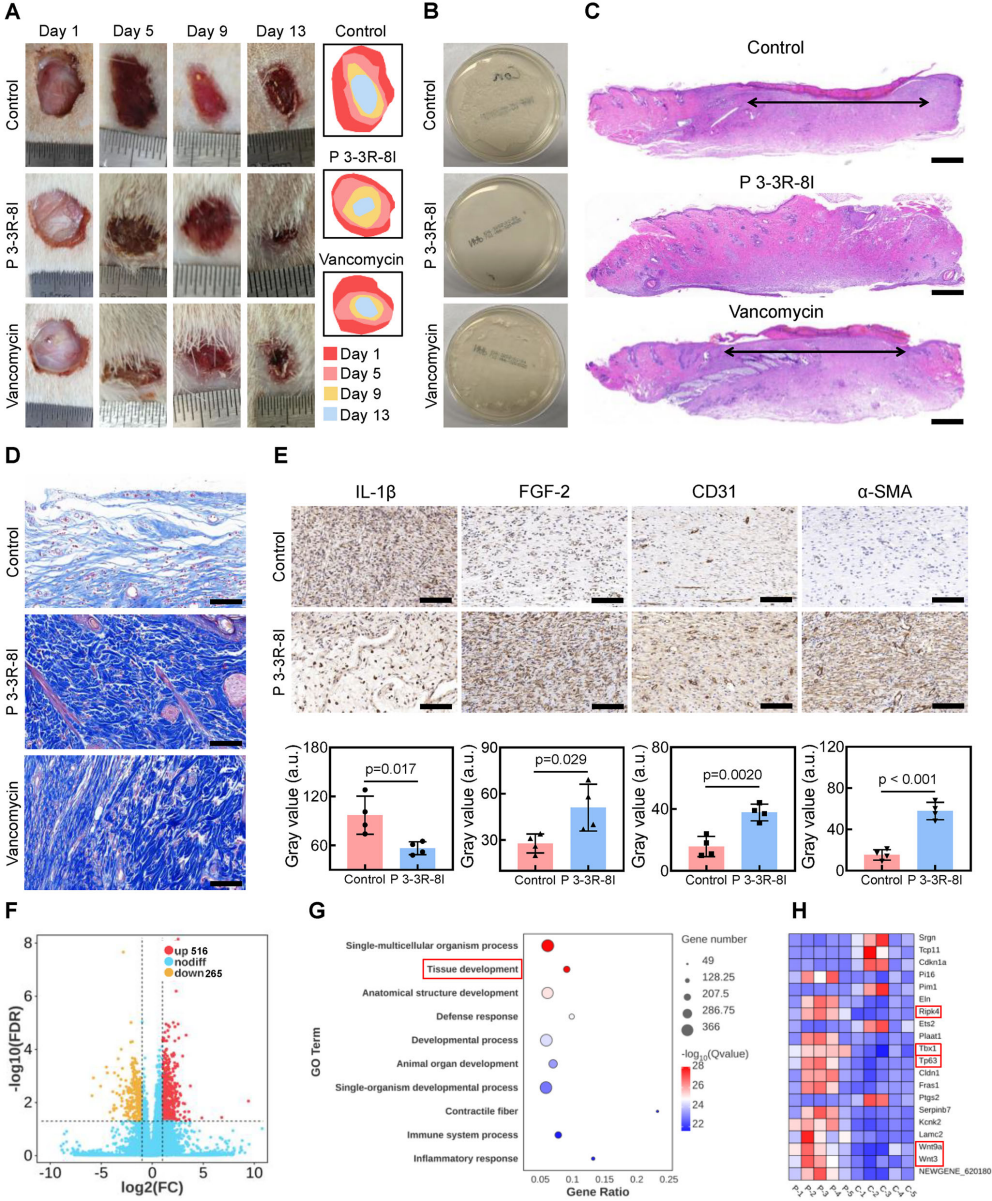
**P 3‐3R‐8I** accelerates the MRSA‐infected wound healing. (A) The representative photograph of wound states and wound traces in different groups within 13 days. n = 5. (B) The representative bacterial colonies of skin tissues from different groups on the culture plate on day 13. n = 5. (C) The representative H&E staining images of skin tissues on day 13. n = 3. Scale bar: 500 µm. (D) The representative images of masson staining in different groups on day 9. Blue: collagen. n = 3. Scale bar: 20 µm. (E) Up: the representative immunohistochemical staining images of IL‐1β, FGF‐2, CD31, and α‐SMA. Scale bar: 20 µm. Down: Statistics of gray values of corresponding images, and higher gray values mean higher expression of factors. n = 4. (F) Differential gene volcano map represented the number of up‐regulated or down‐regulated gene expressions between control and **P 3‐3R‐8I** groups. (G) Enrichment analysis of GO between different samples. (H) Differential gene clustering heat maps of different samples (C: control group, P: **P 3‐3R‐8I** group). The blue means a lower abundance of RNA, and red means a higher abundance of RNA. n = 3.

Considering the antibacterial performances of P 3‐3R‐8I, the application of it in wound healing management was explored. Before that, the in vitro and in vivo biocompatibility of P 3‐3R‐8I was examined. As shown in Figure S23, P 3‐3R‐8I exhibited no effects on the cell viabilities against HUVEC and SVEC4‐10 cells. Furthermore, no damages or inflammations were observed in the major organs of Sprague‐Dawley rats (SD rats) treated with P 3‐3R‐8I (1.00 and 4.00 mg/kg, once every 3 days, 3 times, Figure S24A), suggesting the biosecurity of it. In addition, the effects P 3‐3R‐8I on the concentrations of immunoglobulin M (IgM) and immunoglobulin G (IgG) in rats’ serums were measured to evaluate the immunogenicity of P 3‐3R‐8I. As shown in Figure S24B, the concentrations of IgM and IgG in serums from rats treated with P 3‐3R‐8I are almost unchanged compared to the control group, indicating the immune responses of rats were not affected by P 3‐3R‐8I. The pharmacokinetics of P 3‐3R‐8I in rats’ serums was studied. As illustrated in Figure S25, the half‐elimination time (t_1/2_) of P 3‐3R‐8I in rats’ serums was about 5.7 h, which was longer than that of Vancomycin (about 3.5 h) [35]. This result will provide us with the basis for medication. Moreover, the stability of antibacterial ability of P 3‐3R‐8I in serum is crucial for its in vivo applications, [18, 36] the antibacterial performances of P 3‐3R‐8I after treatment with rats’ serum was evaluated. As shown in Figure S26, P3‐3R‐8I incubated with rats’ serum for 8 h still exhibited stable antibacterial abilities, exhibiting its great potential in in vivo applications. Then, SD rats were randomly divided into three groups: control, P 3‐3R‐8I and Vancomycin (positive control [37]) groups. The wounds in the control group were infected with MRSA and phosphate buffered saline (PBS) was used for treatment. The infected wounds in P 3‐3R‐8I and Vancomycin groups were treated with P 3‐3R‐8I (0.17 mg/kg, once every 3 days, 3 times, the dosage selection principle is noted in the experimental section) and Vancomycin (0.50 mg/kg, once every 3 days, 3 times, the dosage selection principle is noted in the experimental section). As shown in Figure 4A, more substantial degrees of wound closure were observed in rats treated with P 3‐3R‐8I (0.17 mg/kg) or Vancomycin (0.50 mg/kg) compared with the control group. The AMR [38] and toxicities [39, 40] of Vancomycin make it as an emerging threat to public health, weakening its broader applications. On the contrary, P 3‐3R‐8I with no side effects demonstrated great potential in MRSA‐infected wound healing management. To confirm the antibacterial performance of P 3‐3R‐8I in vivo, the tissues were collected for antimicrobial evaluations. As illustrated in Figure 4B, the areas of bacterial colonies from recovered wound tissues treated with P 3‐3R‐8I were smaller than those of other groups. At the same time, granulation tissues were observed in the control and Vancomycin groups, while almost no granulations existed in the P 3‐3R‐8I group (Figure 4C), suggesting the wound treated with P 3‐3R‐8I had a faster healing speed. In addition, collagen played critical roles in wound healing, [41] masson staining results exhibited that more collagen fibrils were formed or gathered in the recovered wound tissues treated with P 3‐3R‐8I or Vancomycin (Figure 4D).

Except for H&E staining, immunohistochemical staining (IHC) was carried out to evaluate some important factors which are related to the wound healing process. Compared to the control group, the **P 3‐3R‐8I** group exhibited less expression of Interleukin‐1β (IL‐1β) (Figure 4E), confirming the distinctive bactericidal role of **P 3‐3R‐8I**. Meanwhile, endothelial cell proliferation, angiogenesis, and wound closure could be promoted by fibroblast growth factor 2 (FGF‐2), [42, 43] hence, the higher expression of FGF‐2 implied a faster wound healing rate. In addition, it is reported that the formation of fresh blood vessels was mediated by platelet endothelial cell adhesion molecule (CD31) and alpha‐smooth muscle actin (α‐SMA). [44] So the higher expressions of CD31/α‐SMA in the **P 3‐3R‐8I** group mean a greater vessel density in skin tissue, which is beneficial to wound healing.

To further investigate the potential biological mechanism of wound healing induced by **P 3‐3R‐8I**, whole genome RNA expression sequencing (RNA‐seq) in rats’ skin tissues was conducted, and the Gene Ontology (GO) enrichment analysis was performed to elaborate the biological process. As shown in Figure 4F, 516 genes were up‐regulated, and 265 genes were down‐regulated in **P 3‐3R‐8I** group compared to the control group. The differential genes were main enriched in several physiological processes such as tissue development, animal organ development, and inflammatory response, etc (Figure 4G). Among them, tissue development is related to the wound healing process, and the up‐regulated or down‐regulated genes were summarized in the gene cluster heat map (Figure 4H). Receptor‐interacting serine/threonine kinase 4 (RIPK4) is a key regulator of wound repair, [45] its up‐regulation was closely related to accelerated wound healing. [46] Likewise, T‐box transcription factor (Tbx1) was essential in forming tissues. [47] In addition, wound healing processes could be promoted by tumor protein p63 (Tp63), [48] Our results showed that Tp63 was up‐regulated upon treatment of **P 3‐3R‐8I** treatment, suggesting the **P 3‐3R‐8I**‐induced faster wound healing process. Moreover, the wound healing could be enhanced by Wnt signal pathways, [49] the higher expressions of Wnt9a/Wnt3 (Figure 4H) in the **P 3‐3R‐8I** group were consistent with the literature results. All these results demonstrate that our AMP: **P 3‐3R‐8I** is an ideal candidate for MRSA‐infected would healing management.

### 2.5 The Therapeutic Effects of P 3‐3R‐8I on MRSA Infection in a Systemic Sepsis Model

**FIGURE 5:**
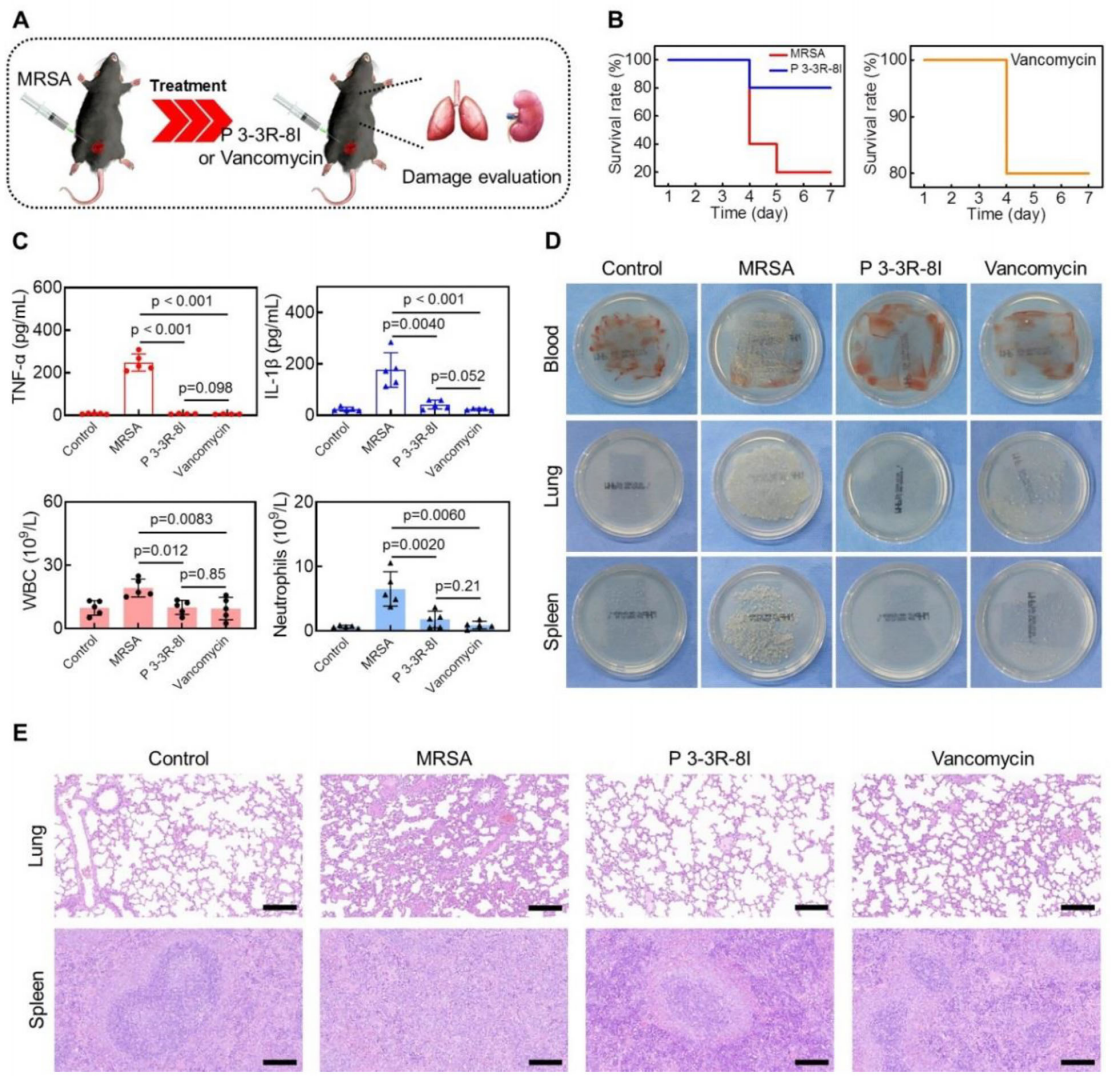
**P 3‐3R‐8I** alleviates infections and damages of lungs and spleens in a systemic sepsis model. (A) The schematic diagram of systemic sepsis construction and treatment. (B) The rats’ survival curves from different groups. n = 5. (C) The concentrations of TNF‐α, IL‐1β, and the counts of WBC and neutrophils in the rats’ serums from different groups. n = 5, *note*: one TNF‐α concentration in P 3‐3R‐8I and Vancomycin group is below the detection limit. (D) The representative bacterial colonies of blood, lung, and spleen from different groups on the culture plate. n = 5. (E) The representative H&E staining images of the lung and spleen. n = 3. Scale bar: 20 µm. n = 3.

In addition to the above wound healing management study, we also investigated the therapeutic effects of **P 3‐3R‐8I** on MRSA infection in a systemic sepsis model. MRSA was intraperitoneal injected into rats to construct the sepsis model, and **P 3‐3R‐8I** or Vancomycin was injected (1.0 mg/kg, once every 2 days, 3 times, the dosage selection principle is noted in the experimental section) to evaluate the infection and damage states (Figure 5A). The survival rates of rats were improved by **P 3‐3R‐8I** or Vancomycin obviously (Figure 5B, 80% to 20%). In addition, the concentrations of tumor necrosis factor‐α (TNF‐α) and interleukin‐1 beta (IL‐1β), and the counts of white blood cell (WBC) and neutrophils in the serums from the treated group were lower than those of infected group (Figure 5C). The infections of blood, lung, and spleen were greatly relieved by **P 3‐3R‐8I** (Figure 5D). Moreover, the damage levels in lungs and spleens from the treated group were significantly lighter than infected group (Figure 5E). All these results show that **P 3‐3R‐8I** has great application potential in the treatment of systemic sepsis.

## 3 Conclusion

In summary, an AMP (**P 3‐3R‐8I**) derived from insect cuticle targeting bacterial cell membranes has been precisely constructed via the amino acid mutation method. **P 3‐3R‐8I** exhibits super antibacterial capability against two representative bacteria: MRSA and *E. coli* (ATCC 25922), which could be attributed to the ability of **P 3‐3R‐8I** to quickly penetrate bacterial cell membranes and then to bind to bacterial DNA, resulting in the suppression of DNA replication. In vivo, the MRSA‐infected wound could be alleviated by **P 3‐3R‐8I** obviously, as well as lung and spleen infections in MRSA‐induced systemic sepsis. Our findings highlight a prospect for precise construction of AMPs targeting bacterial cell membranes based on natural peptides as well as a means of overcoming AMR, offering a strategy for drug‐resistant bacteria‐induced tissue repair.

## 4 Experimental Section

### 4.1 Reagents, Strain and Antibodies

Drug susceptibility inoculation culture (Fluid turbidity type, Cat. No.: G10251) was purchased from Kangtai Biotechnology Co. Ltd., Wenzhou, China. Magnetic bead bacterial‐based genomic DNA extraction kit (Cat. No.: MPC2412043) was purchased from Servicebio. Co., Ltd. MRSA (clinical isolation), polymyxin‐resistant *E. coli* (clinical isolation) and polymyxin‐resistant *K. pneu* (clinical isolation), and *E. coli* (ATCC 25922) were obtained from the Department of Laboratory Medicine, the First Affiliated Hospital of Xi'an Jiaotong University. Peptides were synthesized from Shanghai Apeptide Co. Ltd., China. DiSC3(5) fluorescent probe (CAS No.: 53213‐94‐8, Cat. No.: HY‐D0085), Meropenem (CAS No.: 96036‐03‐2, Cat. No.: HY‐13678) and Vancomycin (CAS No.: 1404‐90‐6, Cat. No.: HY‐B0671) were purchased from MedChemExpress. Agarose (Cat. No.: 1110GR100) was purchased from Biofroxx. SafeRed Loading Dye (Cat. No.: D013) was purchased from ABP Biosciences. DL2000 DNA Marker (Cat. No.: TSJ011‐500) was purchased from TSINGKE. Nitrophenyl‐β‐d‐galactopyranoside (ONPG, Cat. No.: N1127) probe was purchased from Merck, USA. Viability/cytotoxicity assay for bacteria live & dead cells (Cat. No.: 40274ES60) was purchased from YEASEN Co. Ltd. Anti‐IL‐1β polyclonal antibody (Cat. No.: 26048‐1‐AP), anti‐FGF‐2 polyclonal antibody (Cat. No.: 11234‐1‐AP), anti‐CD31 polyclonal antibody (Cat. No.: 33075‐1‐AP), anti‐α‐SMA specific monoclonal antibody (Cat. No.: 67735‐1‐Ig), rat IL‐1 beta Elisa kit (Cat. No.: KE20021) and rat TNF‐α Elisa kit (Cat. No.: KE20018) were purchased from ProteinTech. QuantiCyto rat IgG Elisa kit (Cat. No.: ERC016.96) and QuantiCyto rat IgM Elisa kit (Cat. No.: ERC017.96) were purchased from NEOBIOSCIENCE. Cell counting kit‐8 (CCK‐8, Cat. No.: C0038) was purchased from Beyotime. Other reagents or chemicals were local.

### 4.2 Peptide Structure Prediction

The peptide sequences were submitted to the AlphaFold3 website (https://alphafoldserver.com) for structural prediction. Subsequently, the predicted peptide structures were visualized using ChimeraX software.

### 4.3 ^1^H NMR Spectrum

The spectrum was obtained from Shanghai Apeptide Co. Ltd., China. Via Bruker Avance III 600 MHz NMR spectrometer.

### 4.4 CD Spectrum

CD spectra of the **P 3‐3R‐8I** solution (250 µM, pH 7.40) were collected using an AVIV 420 CD spectrometer (New Jersey, USA). A quartz sandwich cuvette with optical path length of 0.2 mm was used for all data collection. Data were acquired over a wavelength range of 180–260 nm, and acquisition parameters were 1.0 nm wavelength steps with an averaging time of 0.1 s, and readings were averaged over three scans. The secondary structures of **P 3‐3R‐8I** was calculated from https://bestsel.elte.hu/results.php.

### 4.5 t‐SNE Clustering and Methods

To represent the intrinsic relationships between amino acids within a particular peptide, the Word2Vec language framework was trained on short peptides (less than 50 amino acids) curated from UniProt database by setting “subcellular location” filter. The methods of skip‐gram and “hierarchical softmax” were selected, with parameters of context window, vector size for each amino acid, and minimum word count setting as 50, 100, and 1, respectively. Subsequently, huge amounts of experimentally verified AMP sequences (n = 8,860) were collected from public databases, and were mathematically represented by Word2Vec methods. The statistical model of t‐distributed stochastic neighbor embedding (t‐SNE) 6 was used to visualize the high‐dimensional data of peptide Word2Vec embeddings (n = 8,861, including **P 3‐3R‐8I** of interest), with graphs created by using MATLAB R2024a. The primary task of t‐SNE is to assess and retain the probabilistic differences between all data in low‐dimensional space. The original codes for creating t‐SNE graph and the pre‐trained models of the Word2Vec language framework have been uploaded to https://github.com/ChenSizhe13893461199/t‐NSE_AMP_codes.

### 4.6 In Vitro Antibacterial Assays

The MCR properties of MRSA, *E. coli* (ATCC 25922), polymyxin‐resistant *E. coli* (clinical isolation), and polymyxin‐resistant *K. pneu* were identified by TsingkeBiotechnology Co.,Ltd. MRSA, *E. coli* (ATCC 25922), polymyxin‐resistant *E. coli* (clinical isolation) or polymyxin‐resistant *K. pneu* (1.5 × 10^8^ CFU/mL) were diluted 1000 times, and then 50 µL of the strain liquid was added into 96 well plates. Then 50 µL of peptide, Meropenem, Vancomycin solutions or PBS were added (The final concentrations of peptides have been set). The 96 well plates were incubated at 37°C for 24 h. The absorbance values at 630 nm (OD 630) of each well were recorded using a BioTek Synergy LX multimode microplate reader (Agilent Technologies, USA). Then, 50 µL of the resulting strain suspensions were spread on the surface of LB agar medium (Guangzhou Dijing Microbial Technology Co. Ltd. China), and the mediums were incubated at 37°C for 24 h and then photographed.

### 4.7 Live and Dead Bacterial Staining

The MRSA or *E. coli* (ATCC 25922) suspensions were prepared according the previous protocols. Then, 100 µL of suspensions were added into 1 µL of 100 × dye solutions (Solutions containing 1 volume of DMAO, 2 volumes of EthD‐III, and 8 volumes of 0.9% NaCl) and incubated for 20 min without light. The fluorescent images were obtained using a Leica TCS SP5 laser confocal microscope (Leica, Germany).

### 4.8 ONPG Assays

The MRSA or *E. coli* (ATCC 25922) suspensions were prepared and then 100 µL of the suspensions was added into 100 µL of ONPG solutions (Finally concentration: 200 µM) and incubated for 9 h at 37°C. The OD 450 values were recorded using a BioTek Synergy LX Multimode Reader.

### 4.9 SEM Assays

The MRSA or *E. coli* (ATCC 25922) suspensions were prepared, and then the suspensions were centrifuged at 3000 rpm/min for 10 min. The supernatants were discarded, and the strain precipitations were added with 1 mL of 2.5% glutaraldehyde slowly. The precipitations were dispersed carefully, fixed, and stored at 4°C overnight. The fixed solutions were washed with ultrapure water and then fixed with 1% osmium tetroxide for 1 h. After that, the samples were dehydrated and transferred to silicon substrates for conductive treatment. The SEM images were recorded through a JSM‐IT700HR (JEOL, Japan) SEM equipment.

### 4.10 MD Simulations

The structures of gram‐positive bacterial‐mimicking membranes (POPG:POPE = 3:1) and gram‐negative bacterial‐mimicking membranes (POPG:POPE = 1:3) were constructed through CHARMM‐GUI software. The POPG and POPE molecules were described through CHARMM36 force field. The water molecules were described through TIP3P models. All ion concentrations were set to the physiological ones. The energies of systems were minimized (1000 kJ/mol/nm) via the steepest descent method. Then Then a 2‐step NVT hot bath (298.15 K) with a total of 500 ps was used, followed by 4 steps of 1.75 ns kinetic pre‐equilibration, and 500 ns of simulations were finally performed. Temperature coupling was realized via a V‐rescale temperature controller, and pressure‐temperature coupling was realized via a C‐rescale pressure controller, and the pressure is set to 1 bar. Long‐range van der Waals interactions were cut off at 1.2 nm. The particle‐mesh Ewald was used to handle the electrostatic interactions. The simulation step was set as 2fs, and the traces were recorded every 20 ps. All simulations were applied with periodic boundary conditions, including x, y, and z directions.

### 4.11 Molecular Docking

The structures of *Staphylococcus aureus*s’ DNA (Sequence: NC_007795.1, *Escherichia coli*s’ DNA (Sequence: NC_000913.3), and **P 3‐3R‐8I** were predicted through AlphaFold3 website (https://alphafoldserver.com), and the most stable predicted structures were used for molecular docking. The molecular docking was performed via HDOCK software. The docking scores were calculated through iterative scoring function: ITScorePP or ITScorePR.

### 4.12 DiSC3(5) Fluorescent Membrane Probe Assay

MRSA or *E. coli* (ATCC 25922) (1.5 × 10^8^ CFU/mL) solutions were incubated with DiSC3(5) probe (5 µM) at 37°C for 1 h without light. Then 2.5×MIC of **P 3‐3R‐8I** was added into the solutions, and the solutions were incucated for different time (0‐60 min) at 37°C. The fluorescence was recorded via a Spark multifunctional microplate reader (E_x_ = 622 nm, E_m_ = 670 nm).

### 4.13 EMSA

DNA was extracted using a magnetic bead bacterial‐based genomic DNA extraction kit. The concentrations of DNA were determined via an ultra‐micro‐volume spectrophotometer. The extracted DNA was incubated with **P 3‐3R‐8I** (0.25×MIC or 2.5×MIC) for different time at 37°C. Then the loading quality of each tunnel was set as 200 ng. The electrophoresis was performed on 1% agarose gel at 100 V for 40 min, and the gels were placed to a gel documentation imaging system (JY04S‐3C, Beijing JUNYI Electrophoresis Co. Ltd.) and photographed.

### 4.14 DNA Electrophoresis

The MRSA or *E. coli* (ATCC 25922) suspensions were prepared, and then the DNA was extracted using a magnetic bead bacterial‐based genomic DNA extraction kit. The concentrations of DNA were determined via an ultra‐micro‐volume spectrophotometer. 100 mL of 2% agarose gel was prepared. 5 µL of extracted DNA samples (the loading quality of each tunnel was set as 250 ng) was added with 1 µL of SafeRed Loading Dye, and then the solutions were injected into 2% agarose gel. The gels were placed to the electrophoresis instrument and electrophoresed for 20 min (Voltage: 140 V, current: 280 mA). Lastly, the gels were placed to a gel documentation imaging system (JY04S‐3C, Beijing JUNYI Electrophoresis Co. Ltd.) and photographed.

### 4.15 In Vitro Biosecurity

HUVEC and SVEC4‐10 cells were cultured into 96 well plates (1 × 10^5^ cells/mL). Cells were treated without or with **P 3‐3R‐8I** (0.1, 0.5, and 1 mM) for 24 h at 37°C. Then, cell viabilities were measured according to the CCK‐8 protocols. The absorbance value of each well was recorded through the BioTek Synergy LX multimode micro‐plate reader (Agilent). The cell viabilities of cells treated without **P 3‐3R‐8I** were set as 100%.

### 4.16 Serum Stability of **P 3‐3R‐8I**

The serums were collected from rats. **P 3‐3R‐8I** (250 µM) was incubated with serum at 37°C for 4 and 12 h. Then the effects of **P 3‐3R‐8I** on the bacterial survival rates were performed according to the previous protocols.

In Vivo *Biosecurity, Pharmacokinetics and MRSA‐Infected Wound Healing Evaluation*: All animal experimental procedures were performed in accordance with protocols approved by the institutional animal ethics committee of Xi'an Jiaotong University (Approval No. XJTUAE2025‐1382 and XJTUAE2025‐3658) and laboratory animal administration rules of China. For biosecurity evaluation of **P 3‐3R‐8I**, male SD rats (6‐8 weeks old, 150–200 g) were randomly divided into 2 groups: control and **P 3‐3R‐8I** groups (n = 6). Rats in **P 3‐3R‐8I** group were intraperitoneal injected with **P 3‐3R‐8I** (1.0 or 4.0 mg/kg, 100 µL solutions) on day 1, 4, 7. Rats in control group were intraperitoneal injected with 100 µL of PBS on day 1, 4, 7. All rats were sacrificed on day 9, and the main organs were sliced for H&E staining. For immunogenicity assays, male SD rats (6‐8 weeks old, 150–200 g) were randomly divided into 2 groups: control and **P 3‐3R‐8I** groups (n = 3). Rats in **P 3‐3R‐8I** group were intraperitoneal injected with **P 3‐3R‐8I** (1.0 or 4.0 mg/kg, 100 µL of solutions) on day 1, 3, 5, 7, 9. Rats in control group were intraperitoneal injected with 100 µL of PBS on day 1, 3, 5, 7, 9. All rats were sacrificed on day 14, and the serums were collected for IgG and IgM concentration detection. For pharmacokinetics assay, 3 male SD rats (6‐8 weeks old, 150–200 g) were intravenous injected with FITC‐Ahx‐**P 3‐3R‐8I** (1.0 mg/kg, 50 µL of solutions). Then 100 µL of serums were collected at hour 0, 0.083, 0.167, 0.25, 0.33, 0.5, 1.0, 2.0, 4.0, 12.0, 24.0, and 36.0. The fluorescence was recorded via a Spark multifunctional microplate reader (E_x_ = 490 nm, E_m_ = 520 nm). The relative content of **P 3‐3R‐8I** was calculated according to the following formula:

$$\mathrm{Relative}\;\mathrm{content}\;\left(\%\right)=\frac{\mathrm{Ft}}{F0}\times 100\%$$

where *Ft* is the fluorescence at a certain time, *F0* is the fluorescence at 0 h. For MRSA‐infected wound healing evaluation, male SD rats (6‐8 weeks old, 150–200 g) were randomly divided into 3 groups: control (MRSA infection), **P 3‐3R‐8I** treating, and Vancomycin treating groups (n = 8). Circular full‐thickness wounds with diameters of 10 mm were made on the back of the rats, and the wounds were infected with MRSA (1×10^6^ CFU/mL, 50 µL of solutions) for 12 h. The wounds in control group were smeared with PBS (50 µL) on day 1, 5, and 9. Wounds in **P 3‐3R‐8I** and Vancomycin groups were smeared with **P 3‐3R‐8I** (0.17 mg/kg, 50 µL of solutions) and Vancomycin (0.5 mg/kg, 50 µL of solutions) on day 1, 5, and 9, respectively. Note: the basis of **P 3‐3R‐8I** in vivo dosage for wound management is that: (250 µMM‐**P 3‐3R‐8I** × 50 µL) × 2721 (molecular weight)/200 g (rat average weight) = 0.17 mg/kg. According to our previous experience, the dosage of Vancomycin for in vivo antibacterial was 0.5‐5.0 mg/kg for rats. (22) In order to reduce the possible toxic side effects of Vancomycin, 0.5‐1.0 mg/kg Vancomycin (higher than **P 3‐3R‐8I**) may be favorable, which was consistent with its shorter t_1/2_ value. The wound sizes were recorded correspondingly. After treatment, rats were sacrificed on day 13, and the wound tissues were sliced for H&E, Masson, and IHC staining (Servicebio). The strains were cultured on LB agar mediums. The RNA samples were collected for RNA‐seq assay (GENE DENOVO Co. LTD., Guangzhou).

### 4.17 RNA‐Seq

RNA‐seq results were obtained from GENE DENOVO Co. LTD., Guangzhou, China.

### 4.18 Systemic Sepsis Evaluation

Male SD rats (6‐8 weeks old, 150–200 g) were randomly divided into 4 groups: control, MRSA infection, **P 3‐3R‐8I** treating, and Vancomycin treating groups (n = 5). Rats in MRSA infection group were intraperitoneal injected with MRSA (1 × 10^8^ CFU/mL, 2 mL of solutions). Rats in **P 3‐3R‐8I** treating group were intraperitoneal injected with **P 3‐3R‐8I** (1.0 mg/kg, 2 mL of solutions) on day 1 (12 h after MRSA injection), 3, 5. Rats in Vancomycin treating group were intraperitoneal injected with Vancomycin (1.0 mg/kg, 2 mL of solutions) on day 1 (12 h after MRSA injection), 3, 5. Note: due to that the MRSA infections in the systemic sepsis model were multi‐organ types, the dosages of Vancomycin and **P 3‐3R‐8I** were increased. Rats in the control group were intraperitoneal injected with 2 mL of PBS. The rats’ survival curves were recorded every day. On day 7, all living rats were sacrificed, and the serums and lungs, and spleens were collected (for dead rats, samples were collected in advance). The serums were used for TNF‐α concentrations, IL‐1β concentrations, WBC counts, and neutrophil counts measurements. The lung and spleen were sliced for H&E staining and were minced for strain incubations. The blood was also collected for strain incubations.

### 4.19 Statistical Analysis

Statistical analysis was performed using GraphPad Prism 9.0 software. All data are determined from at least three independent experiments and presented as mean ± standard deviation. Statistical significance between multiple groups was assessed by ANOVA followed by Dunnett post‐hoc test (ANOVA Dunnett't test), and statistical significance between two groups was assessed by two‐tailed student's t test. p<0.05 was considered statistically significant.

## Author Contributions

J. Q. Huang and B. H. Liu contributed equally to this work. J. Q. Huang contributed to conceptualization, methodology, and original draft preparation. J. Q. Huang, B. H. Liu, X. Z. Zhu, and D. Q. Qian performed experiments. S. Z. Chen contributed to the MD simulation results analysis. X. Y. Zeng and Q. Q. Yang provided strains. Z. H. Wei contributed to editing. Y. J. Huang and Q. Y. Gong contributed to the review. J. Z. Wang, G. J. Zhang, and Q. Y. Gong provided supervision and acquired funding.

## Funding

National Natural Science Foundation of China (Grant No. 82470107), Capacity Improvement Plan of Shaanxi Health Committee (Grant No. 2024PT‐09), Key Research and Development Project of Shaanxi Province (2023‐YBSF‐292), Key Research and Development Project of Shaanxi Province (2025SF‐YBXM‐344), Opening Foundation (M2022‐3) from the Key Laboratory of Optic‐Electric Sensing and Analytical Chemistry for Life Science, Ministry of Education, Qingdao University of Science and Technology.

## Conflicts of Interest

The authors declare no conflict of interest.

## Supporting information




**Supporting File**: advs73810‐sup‐0001‐SuppMat.docx.

## Data Availability

The data that support the findings of this study are available from the corresponding author upon reasonable request.
