# Supplementary material for: Precise Construction of an Antimicrobial Peptide Targeting Bacterial Cell Membranes Derived From Natural Peptides
Source: Adv Sci (Weinh). 2026 Jan 12;13(17):e17068. doi: 10.1002/advs.202517068 (PMC13042684; doi:10.1002/advs.202517068)
Supplement: Supplementary file 1 — Supporting File: advs73810‐sup‐0001‐SuppMat.docx. [file ADVS-13-e17068-s001.docx]

Supporting Information

Precise Construction of an Antimicrobial Peptide Targeting Bacterial Cell Membranes Derived from Natural Peptides

Jiaqi Huang, Bohao Liu, Xingzhuo Zhu, Deqian Qiao, Sizhe Chen, Xiaoyan Zeng, Qingqing Yang, Zihuan Wei, Yinjuan Huang, Jizhao Wang^*^, Guangjian Zhang^*^ and Qiuyu Gong^*^

J. Q. Huang, X. Z. Zhu, B. H. Liu, D. Q. Qiao, Z. H. Wei, J. Z. Wang, G. J. Zhang, Q. Y. Gong

Department of Thoracic Surgery, The First Affiliated Hospital of Xi’an Jiaotong University, Xi’an 710061, P. R. China.

Key Laboratory of Enhanced Recovery After Surgery of Integrated Chinese and Western Medicine, Administration of Traditional Chinese Medicine of Shaanxi Province, The First Affiliated Hospital of Xi’an Jiaotong University, Xi’an 710061, P. R. China.

S. Z. Chen

Microbiota I-Center, Department of Medicine and Therapeutics, Chinese University of Hong Kong, Hong Kong SAR, P. R. China.

X. Y. Zeng, Q. Q. Yang

Department of Laboratory Medicine, The First Affiliated Hospital of Xi’an Jiaotong University, Xi’an 710061, P. R. China.

Y. J. Huang

State Key Laboratory of Porous Metal Materials, Shaanxi International Research Center for Soft Matter, School of Materials Science and Engineering, Xi'an Jiaotong University, Xi'an, 710049, P. R. China.

Q. Y. Gong

Key Laboratory of Optic-Electric Sensing and Analytical Chemistry for Life Science, Ministry of Education, Qingdao University of Science and Technology, Qingdao 266042, P. R. China.

E-mail: wangjz1104@xjtu.edu.cn; michael8039@xjtu.edu.cn; gongqiuyu@xjtu.edu.cn.

Figure S1. The amino acid sequence of previous antibacterial protein and its peptides.

Figure S2. The HPLC and Mass spectrum of **P 1**.

Figure S3. The HPLC and Mass spectrum of **P 2**.

Figure S4. The HPLC and Mass spectrum of **P 3**.

Figure S5. The HPLC and Mass spectrum of **P 4**.

Figure S6. The HPLC and Mass spectrum of **P 5**.

Figure S7. The W content (%), hydrophobic acid amino residue content (%) and positively charged acid amino residue content (%) in **P 1** to **P 5**.

Figure S8. The photos of E. coli (ATCC 25922) colonies treated with **P 1** to **P 5** (peptide concentrations: 250 μM) for 24 h or without peptides. n=3.

Figure S9. Hydrophilic analysis of **P 1** to **P 5** performed by ProtScale.

Figure S10. The HPLC and Mass spectrum of **P 3-3R**.

Figure S11. The HPLC and Mass spectrum of **P 4-4R**.

Figure S12. The positively charged acid amino residue content (%) in **P 4-4R**, **P 4-4R-5I**, **P 3-3R** and **P 3-3R-8I**.

Figure S13. The HPLC and Mass spectrum of **P 3-3R-8I**.

Figure S14. The ^1^H NMR spectrum of **P 3-3R-8I** (600 MHz, D_2_O-d2, 298 K).

Figure S15. The HPLC and Mass spectrum of **P 4-4R-5I**.

Figure S16. Hydrophilic analysis of **P 3-3R,** **P 4-4R**, **P 3-3R-8I** and **P 4-4R-5I** performed by ProtScale.

Figure S17. Left: the representative photos of MRSA or *E. coli* (ATCC 25922) colonies treated without or with **P 3-3R-8I** (2.5×MIC) for different time. n=3. Right: the turbidities of bacterial liquids treated without or with **P 3-3R-8I** (2.5×MIC) for different time. n=3.

Figure S18. The gene comparison chart of MRSA, *E. coli* (ATCC 25922), polymyxin-resistant *E. coli* and polymyxin-resistant *K. pneu* to MCR-1.

Figure S19. The HPLC and Mass spectrum of FITC-Ahx-**P 3-3R-8I**.

Figure S20. (A) The docking model between **P 3-3R-8I** and gram-positive bacterial-mimicking membranes. (B) The docking model between **P 3-3R-8I** and gram-negative bacterial-mimicking membranes.

Figure S21. The RMSD, RMSF, Rg and CSA of **P 3-3R-8I**-gram-positive bacterial-mimicking membrane system.

Figure S22. The RMSD, RMSF, Rg and CSA of **P 3-3R-8I**-gram-negative bacterial-mimicking membrane system.

Figure S23. The effects of **P 3-3R-8I** on the cell viabilities of HUVEC and SVEC4-10 cells. n=4.


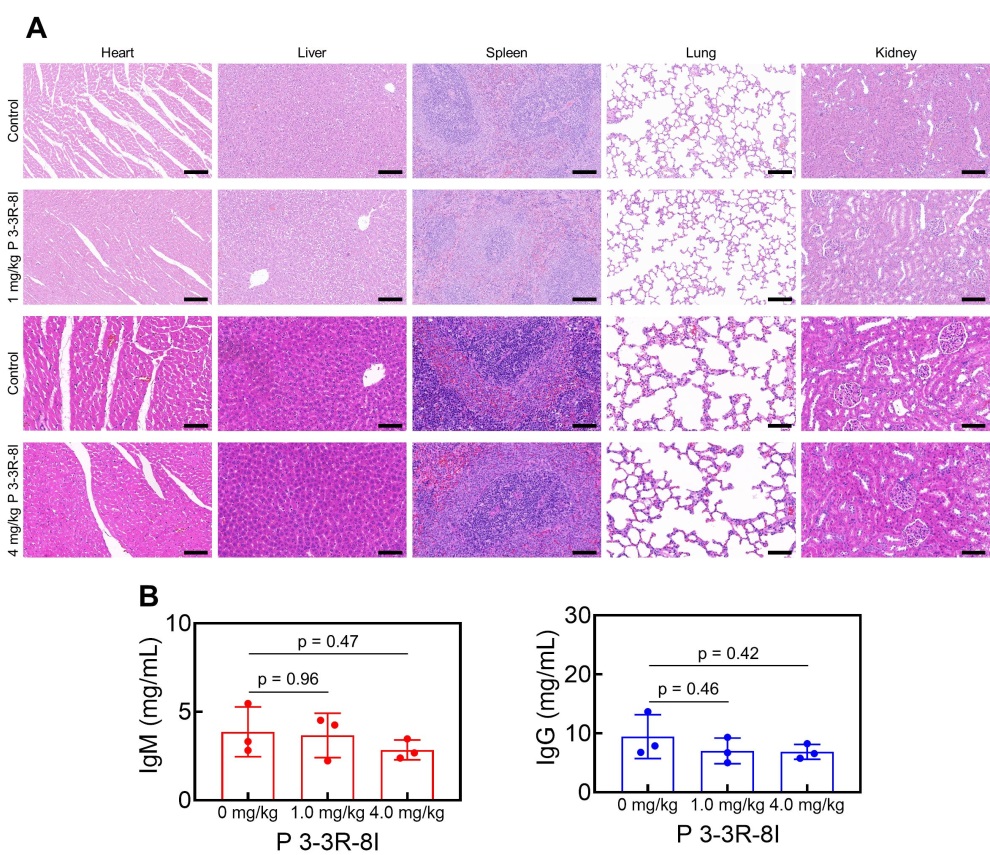


Figure S24. (A) The representative H&E staining images of heart, liver, spleen, lung and kidney from rats in different groups. n=3. Scale bar: 20 μm. (B) The concentrations of IgM and IgG in rats’ serums from different groups. n=3.

Figure S25. The relative contents of FITC-Ahx-**P 3-3R-8I** in rats’ serums with time. n=3.

Figure S26. The representative photos of MRSA or *E. coli* (ATCC 25922) colonies treated without or with **P 3-3R-8I** (2.5×MIC) incubated with rats’ serums for different for 24 hours. n=3.

Table S1. Basic information of **P 1** to **P 5**.

| Peptide | Mw | Iso-electric point (pH) |
| --- | --- | --- |
| **P 1**: SQGWAGPPANIALSQDGRN | 1939.05 | 6.38 |
| **P 2**: WDNEEYWQRAEQPKWNAAP | 2418.53 | 4.04 |
| **P 3**: WNAAPAPSWNAAPANHWNAAP | 2214.36 | 7.69 |
| **P 4**: WNAPAQQQWNAPAPQWNGAP | 2232.37 | 3.7 |
| **P 5**: WQGAPAHQPANIRLAHDGS | 2026.18 | 7.84 |

Table S2. Basic information of **P 3-3R**, **P 4-4R**, **P 3-3R-8I** and **P 4-4R-5I**.

| Peptide | Mw | Iso-electric point (pH) |
| --- | --- | --- |
| **P 3-3R**: WNAAPARRWNAAPARHWNAAP | 2384.62 | 12.4 |
| **P 4-4R**: WNAPARRRWNAPAPRWNGAP | 2344.6 | 12.58 |
| **P 3-3R-8I**: WNIIPIRRWNIIPIRHWNIIP | 2721.26 | 12.4 |
| **P 4-4R-5I**: WNIPIRRRWNIPIPRWNGIP | 2555 | 12.58 |

Table S3. The summary of H bond between **P 3-3R-8I** and *Staphylococcus aureus*s’ DNA.

| **P 3-3R-8I** Residue | Atom name 1 | DNA Residue | Atom name 2 | Distance (Å) | Type |
| --- | --- | --- | --- | --- | --- |
| I20 | O | DA-156(A) | O3’ | 3.2 | H bond |
| R15 | NH1 | DT-250(A) | OP1 | 3.1 | H bond |
| R15 | NH1 | DT-155(A) | OP1 | 2.3 | H bond |
| N18 | N | DA-460(B) | OP2 | 3.3 | H bond |

Table S4. The summary of H bond between **P 3-3R-8I** and *E. Coli*s’ DNA.

| **P 3-3R-8I** Residue | Atom name 1 | | DNA Residue | Atom name 2 | Distance (Å) | Type |
| --- | --- | --- | --- | --- | --- | --- |
| I15 | | O | DC-198(A) | O3’ | 3.2 | H bond |
| W9 | | O | DA-116(A) | O3’ | 2.5 | H bond |
| W9 | | N | DA-117(A) | OP1 | 2.1 | H bond |
